# Unusual Manifestations of Essential Monoclonal Gammopathy. II. Simulation of the Insulin Autoimmune Syndrome

**DOI:** 10.5041/RMMJ.10212

**Published:** 2015-07-30

**Authors:** Marshall A. Lichtman, Sophia R. Balderman

**Affiliations:** 1 Professor of Medicine and of Biochemistry and Biophysics, Department of Medicine and James P. Wilmot Cancer Center, University of Rochester Medical Center, Rochester, NY, USA; 2 Instructor in Medicine, Department of Medicine and James P. Wilmot Cancer Center, University of Rochester Medical Center, Rochester, NY, USA

**Keywords:** Anti-insulin antibodies, hypoglycemia, insulin, insulin autoimmune syndrome, monoclonal gammopathy

## Abstract

In rare cases, the monoclonal immunoglobulin that characterizes essential monoclonal gammopathy interacts with a self-antigen with functional consequences and a resulting clinical syndrome. This event is presumably random and results from the clone of B lymphocytes making a monoclonal immunoglobulin that simulates an autoimmune antibody. Thus, by chance, the monoclonal immunoglobulin has sufficient affinity for an epitope on a normal protein that functional consequences ensue. One such rare event is the synthesis and secretion of a monoclonal immunoglobulin that binds to human insulin. Inactivation of insulin by antibody results in (1) an early postprandial hyperglycemia, (2) followed by either or both (i) a reactive overshot in insulin secretion, as a result of hypertrophied or hyperplastic islet beta cells, later falling glucose levels, and (ii) an unpredictable dissociation of insulin from the complex, and, several hours later, (3) a resultant increase in free insulin levels and severe hypoglycemia with clinical consequences, ranging from sweating, dizziness, headache, and tremors to confusion, seizures, and unconsciousness. These attacks are invariably responsive to glucose administration. This very uncommon manifestation of a monoclonal gammopathy can occur in patients with essential monoclonal gammopathy or myeloma. The monoclonal anti-insulin immunoglobulin in monoclonal gammopathy has a low affinity for insulin, but has a high capacity for insulin-binding, resulting in the syndrome of episodic hypoglycemic attacks. This phenomenon of an insulin-binding monoclonal immunoglobulin simulates the acquired insulin autoimmune syndrome, although the latter is mediated by a polyclonal antibody response in the majority of cases studied, and has linkage to HLA class II alleles.

## INTRODUCTION

Essential monoclonal gammopathy (synonymous with monoclonal gammopathy of unknown significance), which is usually an asymptomatic state, may cause an associated disorder because the monoclonal immunoglobulin is of an aberrant physicochemical structure and (1) can form paracrystalline or crystalline deposits in certain tissue, notably the cornea or the kidney, thereby leading to crystalline keratopathy or a renal impairment syndrome; (2) can be deposited in macrophages resulting in crystal-storing histiocytosis, usually involving organs of the mononuclear phagocyte system, and other tissues, including the orbit; or (3) can exhibit exaggerated copper-binding and deposition of copper in the Descemet membrane, a tissue stratum between the stroma and endothelium of the cornea.^1^

Another type of presumably random event can result from the monoclonal immunoglobulin having sufficient affinity for a biologically active molecule (self-antigen) to induce a condition analogous to classical autoimmune disease in which the autoantibody is provoked by an autoantigen in the setting of loss of tolerance (e.g. acquired von Willebrand disease).^2^ We review the rare cases in which the monoclonal immunoglobulin, acting as an insulin-binding autoantibody, simulates the insulin autoimmune syndrome.

## DISCOVERY OF INSULIN ANTIBODIES AND SEVERE HYPOGLYCEMIC ATTACKS IN PATIENTS WITH ESSENTIAL MONOCLONAL GAMMOPATHY OR MYELOMA

In 1972, a 61-year-old woman manifested episodic confusion, apparently unrelated to other neurological abnormalities. Indeed, while in the hospital under study, she had two episodes of sudden unconsciousness and left-sided paralysis, but within a few hours she had completely recovered; these events were identical to those that led to her admission. Blood sugars of 10 to 19 mg/dL were found at the time of the episodes that occurred while she was hospitalized; the episodes were reversed by glucose administration. Her medical studies subsequently revealed the presence of an IgA-secreting myeloma. The reporting physician did no further studies related to the hypoglycemic episodes, but he implied that this might be a hitherto never-described metabolic abnormality associated in some way with myeloma (Table 1).^3^

**Table 1:** Monoclonal Gammopathy-induced Insulin “Autoimmune” Syndrome.

Citation / Year of Report	Age (y) / Gender (M/F)	Monoclonal Ig Isotype	Insulin Antibodies Kinetics	Evidence
**Essential Monoclonal Gammopathy**				
4 / 1986	63 / M	IgG-kappa	Capacity (estimated): 240×10^−6^ mol L^−1^ Affinity (estimated): *K_a_*=0.2×10^6^ L mol^−1^	Specific binding of monoclonal IgG-kappa to insulin
5 / 1989	64 / M	IgG-lambda	Capacity: 1.7×10^−6^ mol L^−1^ Affinity: *K_a_*=1.6×10^6^ L mol^−1^	Specific binding of monoclonal IgG-lambda to insulin
9 / 1993	48 / F	IgG (light chain type not reported)	Capacity: Not described Affinity: *K_a_*=4.0×10^5^ L mol^−1^	Anti-insulin antibodies identifiable by polyethylene glycol precipitation. ^125^I-insulin binding by autoradiography to monoclonal IgG on agarose gel electrophoretic separation in an amount that can be decreased by unlabeled insulin
10 / 2004	83 / F	IgG-kappa	Capacity 1.9×10^−5^ mol L^−1^ Affinity: *K_a_*=1.4×10^6^ L mol^−1^	Anti-insulin antibody corresponded to the monoclonal IgG
**Myeloma**				
3 / 1972	61 / F	IgA (light chain type not known)	Not studied	Hypoglycemia attacks presenting symptom of myeloma. Posited that the hypoglycemia was in some way related to myeloma
6* / 1990	53 / M	IgG-kappa	Not studied	Disappearance of monoclonal IgG-kappa after radiation of sacral lesion and chemotherapy and coincidental disappearance of hypoglycemic episodes and elevated insulin levels
7 / 1992	73 / M	IgG-lambda	Capacity: 27×10^−6^ mol L^−1^ Affinity: *K_a_*=0.085×10^6^ L mol^−1^	Affinity of IgG-lambda for insulin demonstrated
8 / 1992	78 / M	IgG-kappa	Not studied	IgG-kappa monoclonal immunoglobulin shown to be anti-insulin antibody
11 / 2007	72 / M	IgA-kappa	Capacity 5.7×10^−4^ mol L^−1^ Affinity: *K_d_*=0.32×10^−6^ mol L^−1^	IgA-kappa monoclonal immunoglobulin shown to be a low-affinity, high-capacity anti-insulin antibody
12 / 2012	63 / M	IgG_3_-kappa and IgG_3_-lambda component with insulin-binding capability	Capacity: Not reported Affinity: *K_a_*=7×10^6^ L mol^−1^	Anti-insulin IgG_3_-lambda monoclonal antibody distinct from the primary myeloma clone monoclonal IgG_3_-kappa. Elevated anti-insulin antibody titers and hypoglycemic attacks were closely associated with escape of myeloma from suppression by therapy

* Localized (sacral) myeloma.

Fourteen years later, the first case of hypoglycemia unequivocally associated with insulin antibodies in essential monoclonal gammopathy was reported in a 63-year-old man who had never received insulin.^4^ Over a period of approximately five years, he had episodes of dizziness, excessive sweating, headache, and diminished vision, which increased in severity over that time period. He also had episodes of unconsciousness but learned to avoid these extreme events by taking sugar or a carbohydrate-rich snack at the first sign of onset of symptoms. On medical evaluation, the patient had a restricted gamma globulin band of 3.4 g/dL on agarose gel protein electrophoresis. He had normal values of serum IgA and IgM. Immunoelectrophoresis revealed a monoclonal IgG-kappa immunoglobulin, and marrow contained a monoclonal plasma cell population (10% of marrow cells) of the IgG-kappa type by immunofluorescence. He had no Bence Jones proteins in concentrated urine and no other findings of myeloma (e.g. hypercalcemia or renal or osseous abnormalities). Antibodies to thyroid, smooth muscle, parietal cells, mitochondria, double-stranded DNA, and bile ducts were not found, nor was there evidence of elevated antinuclear or rheumatoid factors. Angiography, computed tomography, and ultrasonography of the pancreas were normal (Table 1).

Radioimmunoelectrophoresis studies established that anti-insulin antibodies were specifically confined to the intact monoclonal IgG-kappa immunoglobulin and not associated with immunoglobulins containing α, μ, or λ chains nor the isolated heavy or light chain of the monoclonal IgG-kappa. Studies during a glucose tolerance test showed a very large amount of fasting antibody-bound insulin and no protein-bound C-peptide. The monoclonal immunoglobulin had a very high binding capacity for insulin as judged by the failure of high concentrations of exogenous insulin to inhibit radioactive insulin binding to the anti-insulin monoclonal immunoglobulin. The severity of the attacks led to treatment to inhibit islet beta cell insulin release with diazoxide, followed by cytotoxic therapy to decrease the clone of IgG-kappa-secreting B lymphocytes. Neither therapy resulted in improvement.

**Figure 1:**
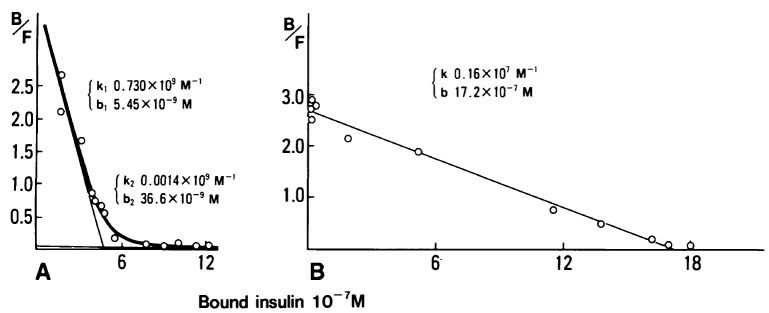
Scatchard Analyses of Equilibrium-binding Assay of Insulin Antibodies; Patient with Insulin Autoimmune Syndrome (A) Compared to a 64-year-old Man Studied Because of Episodes of Hypoglycemia as a Result of an Insulin-binding Monoclonal IgG (B) (A) The patient with insulin autoimmune syndrome has curvilinear response compatible with polyclonal antibodies with a higher and lower affinity binding site. Also, IgG antibodies contained 52.3% kappa light chains and 35.1% lambda light chains. (B) The 64-year-old man with hypoglycemic attacks; his Scatchard plot showed straight-line relationship, suggesting homogeneity of antibody binding site, compatible with a monoclonal anti-insulin antibody. (k, affinity constant; k_1_, higher affinity binding site; k_2_, lower affinity binding site; b, maximum binding capacity; serum dilution 1:45.) (From *Diabetes Care*^5^ and reprinted with permission of the American Diabetes Association. Copyright 1989.)

**Figure 2:**
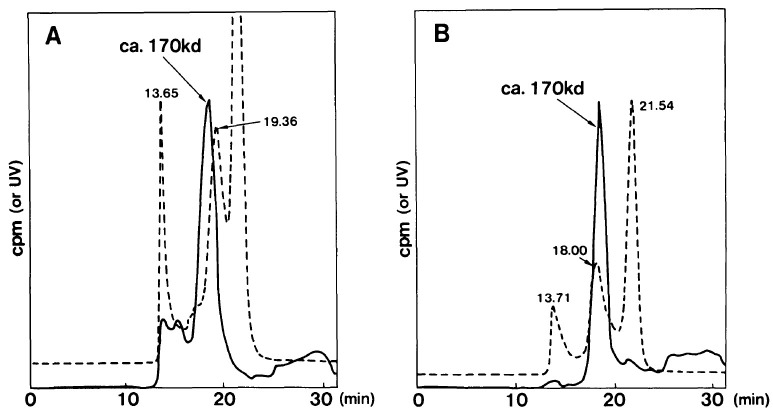
The Elution Profile of ^125^I-labeled Insulin antibody Complex by Molecular Sieve High-performance Liquid Chromatography. Insulin Autoimmune Syndrome compared to Essential Monoclonal Gammopathy The ordinate shows counts per minute (cpm) and ultraviolet absorbance at 280 nM. (A) The patient with insulin autoimmune syndrome (see Figure 1A) had a more heterogeneous elution profile with the main peak accounting for 68.2% of the total radioactive counts. (B) The 64-year-old man with monoclonal gammopathy had a more uniform elution profile. Additional studies indicated the molecular size (*M_r_*) of the IgG insulin complex was 170,000. The IgG was 160,000 and insulin 5,700. Because the IgG had two insulin binding sites the complex was calculated to have a molecular size of 171,400, very similar to the 170,000 identified. (From *Diabetes Care*^5^ and reprinted with permission of the American Diabetes Association. Copyright 1989.)

In 1989, a second case of essential monoclonal gammopathy in a 64-year-old man was found to be responsible for a hypoglycemic syndrome indistinguishable from the insulin autoimmune syndrome.^5^ The patient had episodes of sweating, drowsiness, palpitations, and sometimes grand mal seizures and coma. His blood sugar was <20 mg/dL at the time of these attacks, and the attacks were reversed by intravenous glucose. He had a classical serum IgG-lambda monoclonal protein but no other marrow, chemical, renal, or osseous findings indicative of myeloma (Table 1). The insulin antibodies consisted of monoclonal IgG-lambda, and the Scatchard plot was indicative of a monoclonal antibody (Figure 1). The elution profile of ^125^I-human insulin–antibody complexes using molecular sieve high-performance liquid chromatography eluted virtually as a single peak (Figure 2).

Normal IgG has two binding sites for insulin at saturation, a higher (*K_a_*= 10^10^ to 10^8^ L mol^−1^) and a lower (*K_a_*= 10^8^ to 10^7^ L mol^−1^) affinity site. The cases of monoclonal gammopathy acting as an insulin autoantibody coincide with low-affinity binding, but the monoclonal antibody has a high capacity for binding insulin. The low affinity may permit dissociation of free insulin contributing to the late hypoglycemia, if the binding equilibrium is perturbed.

In 1990, a 53-year-old man with localized myeloma, apparently confined to the sacrum, with normal sternal and iliac crest marrow samples, normal radiographs of other bones, normal blood counts, serum calcium, and kidney function, and an absence of urinary Bence Jones protein presented with hypoglycemic attacks.^6^ A sacral biopsy showed sheets of plasma cells. He had had episodes of sweating, tremulousness, and hunger resolved by eating chocolate or sugar. The attacks occurred multiple times per week. He had an IgG-kappa monoclonal protein (1.7 g/dL) without immunoparesis (normal polyclonal IgG, IgA, and IgM concentrations). In response to a glucose load, the patient had very elevated insulin and C-peptide plasma levels after 3 hours and after a 48 hour fast, using a specific radioimmunoassay. He was treated with radiotherapy to the sacrum, followed by prednisone and melphalan. Subsequent to treatment, within one year of diagnosis, his monoclonal protein disappeared, as did his hypoglycemia. Insulin levels returned to normal (Table 1).

In 1992, a 73-year-old man was described with seizures and unconsciousness as a result of severe hypoglycemia with a response to intravenous glucose.^7^ Recurrent postprandial hypoglycemia, with markedly elevated insulin levels on radioimmunoassay, was evident. The patient had findings in the marrow compatible with myeloma and an IgG-lambda monoclonal immunoglobulin in the serum that was shown by insulin affinity chromatography, elution, and immunoelectrophoresis of the eluate to have bound insulin, specifically. An equilibrium-binding assay of the patient’s serum from which endogenous insulin was depleted resulted in a Scatchard plot that was indicative of a single class of low-affinity, but high-capacity binding sites for insulin (Table 1). That year, an additional case was reported in the German medical literature. The patient, a 78-year-old man, presented with fasting and late postprandial hypoglycemia and markedly elevated levels of immunoreactive insulin.^8^ High titers of insulin antibodies were observed and an insulin autoimmune syndrome diagnosed, but during the medical evaluation a monoclonal IgG-kappa (2.2 g/dL) secreting myeloma was found. The insulin antibodies were shown to be identical to the monoclonal immunoglobulin secreted by his myeloma cells.

**Figure 3:**
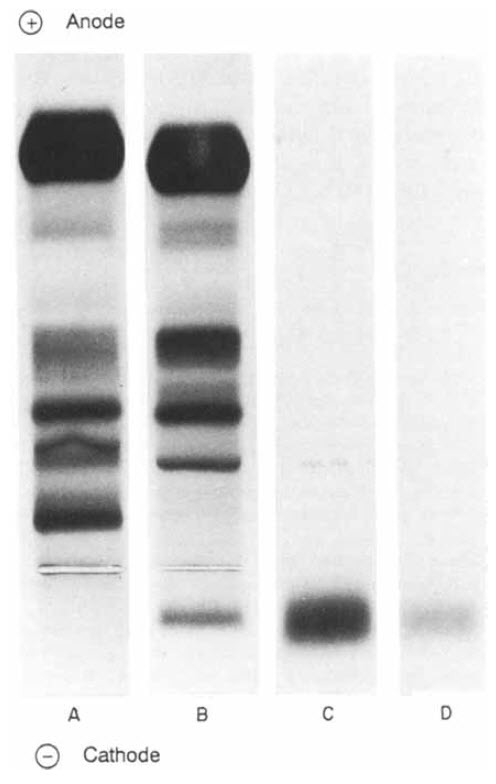
A 48-year-old Woman with Essential Monoclonal Gammpathy Studied Because of Episodes of Hypoglycemia Combined agarose gel electrophoresis (A, B) and autoradiography (C, D). (A) The protein fractionation of normal serum on agarose gel. (B) The patient’s serum indicated a monoclonal IgG at the cathode. (C) A radioautograph showing a heavy band of ^125^I-labeled insulin bound by the monoclonal IgG. (D) The addition of unlabeled insulin markedly reduced the ^125^I-labeled insulin binding to monoclonal IgG. (From *J Intern Med*^9^ and reprinted with permission of John Wiley and Sons.)

In 1993, a third case of a clinical presentation simulating the insulin autoimmune syndrome (*vide infra*) was reported in association with essential monoclonal gammopathy in a 48-year-old woman.^9^ This patient had attacks of severe hypoglycemia two to three hours after breakfast manifested by shivering, sweating, and loss of consciousness. This pattern had been present for about six months. She could relieve her symptoms by eating extra meals. She was observed for three months with continued episodes, and when she lost consciousness again she was hospitalized for evaluation. Her glucose tolerance test showed a normal fasting blood sugar, a postprandial hyperglycemia, and a prolonged, late hypoglycemia. The fasting blood insulin level was abnormally high, as was the proinsulin level. Ultrasonography and computed tomography suggested a pancreatic tumor. She had a pancreatic resection, but no insulinoma was found. Hypoglycemic attacks continued despite removal of 90% of the pancreas. The patient was found to have a monoclonal immunoglobulin IgG in serum on agarose electrophoresis (0.4 g/dL) and 1.1 g/dL of polyclonal IgG (Table 1). The monoclonal IgG was shown to bind insulin by combined agarose gel electrophoresis and autoradiography (Figure 3).

In 2004, an 83-year-old woman was diagnosed with hypoglycemia. She had had episodes of erratic driving, dizziness, and dysphoria relieved by eating. As a result of an auto accident, she was evaluated and found to have episodes of severe hypoglycemia and extraordinarily high total and free insulin and a high-titer anti-insulin autoantibody.^10^ Her serum protein analysis showed a monoclonal IgG-kappa with no Bence Jones protein in urine, a marrow plasma cell frequency of 3%, and no other evidence of myeloma, consistent with essential monoclonal gammopathy. ^125^I-insulin bound to her monoclonal IgG. The insulin–monoclonal IgG binding was of low affinity and high capacity (Table 1).

Three years later, a 72-year-old man was admitted to the hospital with a grand mal seizure and was found to be hypoglycemic and was responsive to intravenous glucose. He had a serum monoclonal IgA of 5.0 g/dL and very low serum IgG and IgM concentrations. An IgA-kappa was identified by immunofixation. His marrow examination and bone imaging resulted in a diagnosis of myeloma, which was treated with four-drug combination therapy.^11^ He had persistent recurrent episodes of severe symptomatic hypoglycemia. Plasma immunoreactive insulin was several hundred times normal during the hypoglycemic episodes. His immunoreactive insulin had a much higher molecular mass than insulin and co-eluted with his monoclonal IgA immunoglobulin. An IgA–insulin complex was identified, and Scatchard analysis showed a single class of binding sites and a low affinity and high capacity for insulin binding by the IgA protein.

In 2012, a study was made of a patient with episodes of blurred vision, diaphoresis, confusion following periods of fasting or late (4–8 h) after meals and relieved by sugar-containing beverages.^12^ These attacks were established to be related to attacks of hypoglycemia. During an observed fast he developed hypoglycemia at six hours with insulin levels 100 times and proinsulin levels 40 times normal (Table 1). Abdominal computed tomography and octreotide scans were normal. Antibodies to insulin were profoundly increased, and a diagnosis of insulin autoimmune syndrome was made. At this time he had elevated serum aspartate and alanine transaminases, and a diagnosis of hepatitis C was made. A liver biopsy showed moderate fibrosis. During this evaluation a monoclonal IgG of 1.5 g/dL was observed on serum protein electrophoresis composed of a dominant kappa and a less prominent lambda fraction, which at the time was presumed to be related to the hepatitis C infection. Several months later, a lytic rib lesion was biopsied and contained a plasmacytoma, and a marrow biopsy had greater than 10% atypical plasma cells. A diagnosis of IgG_3_-kappa-secreting myeloma was made, but a lambda light chain was present, also, on serum immunofixation. The IgG_3_ heavy chain and lambda light chain were insulin-binding, which occurred at position 9 (lysine) on the insulin beta chain. If this residue was removed, binding decreased 3-fold. Heavy and light chains from individual myeloma cells were isolated, and their variable regions were found to be somatically hypermutated, especially in the light and heavy chain complementarity-determining regions. All isolated clones of single myeloma cells had monoclonal IgG with identical variable region sequences. A recombinant monoclonal antibody from cloned heavy and light chains bound insulin but no other self-antigens tested or lipopolysaccharide. The pathogenic, low-affinity anti-insulin antibody was secreted by a clone that had undergone affinity maturation analogous to that occurring in normal germinal centers. The similar sequences indicated similar mutational patterns in heavy and light chains, compatible with an antigen-driven response, but the patient had not had insulin administered previously. A cross-reaction with a protein with a similar structure as the insulin beta chain amino acid residues 15 to 30 was considered, speculatively. The insulin autoimmune syndrome preceded evidence of myeloma by one year. Given the sequence data and clinical expression of the anti-insulin antibody, the authors considered, hypothetically, that the autoreactive plasma cell that produced the anti-insulin antibody, initially, developed normally but later transformed into a plasma cell neoplasm.^12^

## THE INSULIN AUTOIMMUNE SYNDROME

An insulin-binding monoclonal immunoglobulin, as described in the cases of essential monoclonal gammopathy and myeloma above, can simulate the insulin autoimmune syndrome, which has the following four features: (1) recurrent episodes of symptomatic hypoglycemia, sometimes leading to unconsciousness; (2) a response to glucose administration; (3) very high levels of plasma immunoreactive (antibody-bound) insulin; and (4) plasma anti-human insulin antibodies in the absence of prior exposure to insulin.^13^ In most patients, the severity of hypoglycemia and the levels of immunoreactive insulin will decrease over several months, although some patients may have mild residual abnormalities for longer periods.^14^ The symptoms range from dizziness, tremulousness, headache, diaphoresis, lethargy, syncope, seizures, to unconsciousness. Even the occurrence of multiple episodes of reversible, flaccid hemiparesis, responsive to intravenous glucose, has been described.^15^

The disease was first described in 1970 by Hirata and colleagues in Japan.^13^ The syndrome has about 10 times the incidence in East Asia, particularly among Japanese, than in persons in Western Europe based on case reporting.^13^,^16^–^22^ The cases in East Asia (principally Japanese, with some Chinese and Korean subjects) are nearly all the result of polyclonal anti-insulin antibodies, but occasional cases, especially among persons of European descent, have had monoclonal anti-insulin antibodies mediating the syndrome without other evidence of a monoclonal gammopathy.^14^,^15^,^20^,^23^–^25^

Among 582 patients in Great Britain who had an antibody against self-tissue (thyroglobulin, gastric parietal cells, glomerular basement membrane, striated or smooth muscle, nuclear factor, and other sites), nine had anti-insulin antibodies in the gamma globulin fraction of plasma. None were diabetic or had ever received insulin, confirmed by the fact that none had antibodies to the C-peptide of insulin. C-peptide antibodies to bovine insulin were only found in insulin-treated diabetics in one of several comparison groups. Of the nine individuals, four bound human, bovine, and porcine insulin, and five bound only human insulin, at a time when human insulin for administration was not available. Since human insulin differs from porcine insulin by a single amino acid residue, a response to human insulin alone indicated the reaction involved antibodies of high homogeneity, and raised the question of monoclonal anti-insulin antibodies.^26^ Another study of 24 patients with insulin autoimmune syndrome at the Diabetic Center of Tokyo’s Women’s Medical College found all 24 subjects had IgG anti-insulin antibodies but had widely different subclasses of IgG (G_1_, G_2_, G_3_, G_4_) represented, and their subclass distribution was similar to that of a comparison group of healthy Japanese subjects. Only two of the 24 subjects had a single subclass type and one light chain type (one kappa and one lambda), compatible with a monoclonal antibody.^27^ In a later study among Japanese, 50 of 51 subjects had polyclonal anti-insulin antibodies, and 9 of 10 non-Japanese East Asians had polyclonal anti-insulin antibodies.^14^,^28^ Thus, the overwhelming majority of East Asian patients (~97%) with this syndrome have polyclonal antibodies to insulin. Monoclonal anti-insulin antibodies have been examined for the complementary antigen site on the insulin molecule. One such case was found to bind to the B3 residue (asparagine) on the human insulin B chain.^28^

Many patients who were ultimately found to have the insulin autoimmune syndrome have had partial pancreatic excisions or biopsies in an effort to identify an insulinoma. In the cases in which no insulin-secreting tumor was identified, the removed pancreatic tissue has sometimes shown islet beta cell hyperplasia (more beta cells per islet) or hypertrophy (more islets).^15^,^29^

Insulin must be dissociated from its antibody complex for it to exert a hypoglycemic effect. The dissociation constant of antibody-bound insulin in some cases has indicated that sufficient free insulin could be released to produce hypoglycemia. The insulin–antibody complex, unlike normal pancreatic beta cell function, is not subject to a feed-back loop.^30^ The insulin molecule in the insulin autoimmune syndrome is structurally identical to human insulin in non-affected patients.^31^

In 1990, the Japanese reported two surveys of 2,094 hospitals. The years covered were 1979 to 1981 and 1985 to 1987. The results indicated that insulin autoimmune syndrome was the third leading cause of spontaneous hypoglycemia in Japan, accounting for approximately 12% of all cases. Only insulinoma (36%) and extrapancreatic neoplasms (26%) were more frequent, and 13 other causes made up the remaining 26% of cases.^32^

The apparent increase in the insulin autoimmune syndrome in Japan, compared to the frequency of European cases, has led to two possible explanations. First, the association of insulin autoimmune syndrome with Graves disease appeared to be related to the frequent use of methimazole for anti-thyroid therapy in Japan.^32^–^34^ Subsequent surveys suggested that in many cases the syndrome may be related to prior use of drugs that have sulfhydryl groups (e.g. methimazole, glutathione, D-penicillamine).^29^,^32^,^33^ The evidence suggested that T cell immunotolerance, which may prevent insulin autoimmune syndrome despite a predisposing HLA-DR genotype, can be broken if sulfhydryl compounds break S–S bonds in human insulin. This proposition has been supported by redevelopment of the syndrome after second and third challenges with methimazole^33^,^35^ and the ability to enhance, *in vitro*, the proliferative T cell response to insulin by dithiothreitol.^36^

A second explanation for its prevalence was the strong association of HLA-DR4 with the insulin autoimmune syndrome in Japan. Notably, all of 27 patients (100%) who had the insulin autoimmune syndrome studied in Japan had HLA class II alleles, DRB1*0406, DQA1*0301, and DQB1*0302, whereas only 14% of an unaffected comparison group of Japanese carried those antigens.^36^–^39^ In studies of affected and unaffected individuals, these HLA class II alleles were correlated with the response of T lymphocytes to insulin stimulation *in vitro*.^39^ Subsequently, patients with Graves disease who developed insulin autoimmune syndrome after methimazole treatment were shown to carry HLA class II genes with the allelic combination Bww62/Cw4/Dr4, if that combination carried DRB1*0406.^14^,^33^,^40^–^44^ It has been proposed that DRB1*0403 is the ancestral allele of DRB1*0406 and that populations with a higher prevalence of DRB1*0406 have a higher risk of developing insulin autoimmune syndrome; a low incidence of DRB1*0406 among persons of European descent could explain the lower incidence of insulin autoimmunity in the latter population.^24^

Indeed, insulin autoimmune syndrome was the fifth disease to be shown to be tightly linked to an HLA antigen: ankylosing spondylitis with HLA-B27, pemphigus vulgarus with HLA-DR4, narcolepsy with HLA-DR2, primary sclerosing cholangitis with HLA-DRw52a, and insulin autoimmune syndrome (Hirata disease) linked with HLA-DR4.^14^ The dominant phenotype determining susceptibility to insulin autoimmunity is DR4, and DRB1*0406 is associated with the highest risk. Glu-74 in the DR4B1 chain is essential for the polyclonal anti-insulin antibody response. Ser-37 in the DR4B1 chain increases the predisposition to the autoimmune response to insulin.^10^,^20^ Ala-74, instead of Glu-74, in the HLA-DRB1 chain is thought to be key in the production of a monoclonal, rather than a polyclonal, anti-insulin antibody response.^24^ The peptide (^8^TSICSLYQLE^15^) of the human insulin A-chain binds specifically to DRB1*0406.^45^ The DRB1*0403 allele, far more common in persons of European descent, may be associated with the development of monoclonal anti-insulin antibodies.^23^

## RELATIONSHIP OF ESSENTIAL MONOCLONAL GAMMOPATHY TO INSULIN AUTOIMMUNE SYNDROME

The signs and symptoms of both monoclonal gammopathy with an anti-insulin monoclonal immunoglobulin and insulin autoimmune syndrome are indistinguishable. The glucose kinetics and insulin kinetics are also very similar, although the insulin–antibody binding may be stronger, on average, in cases of the insulin autoimmune syndrome. One major difference is that signs and symptoms among Asian cases of the insulin autoimmune syndrome improve and may disappear within several months of onset; only occasional cases persist beyond a year and, if they do, usually with milder signs and symptoms. In the case of monoclonal gammopathy, the treatment of frequent small meals and diets low in simple sugars is required, presumably indefinitely, and patients are educated to self-administer glucose early if symptoms occur, which can prevent severe reactions. In some cases of myeloma, the hypoglycemic attacks were so severe that plasmapheresis, glucocorticoids, and chemotherapy were used to decrease the anti-insulin-binding monoclonal immunoglobulin. In several cases the severity of the hypoglycemia and the level of insulin antibodies closely paralleled the remission–relapse pattern of the myeloma and the level of the monoclonal immunoglobulin.

The precise role of hypersecretion of insulin by primed islet beta cells and of dissociation of the insulin–antibody complex in an individual case of symptomatic hypoglycemia has been studied infrequently. Histopathological examination of pancreatic resections done to search for an insulinoma has shown, in several cases, beta cell hyperplasia or hypertrophy, strongly suggesting that insulin overshoot is one mechanism of the late (4–8 hours postprandial) hypoglycemia. This supposition is supported by one case in which a 90% pancreatectomy had no effect on the hypoglycemic episodes. The low affinity of insulin for the binding antibodies in some cases of the insulin autoimmune syndrome and all cases studied of monoclonal gammopathy sets the stage for a dissociation of the insulin–antibody complex, contributing to an elevated free insulin level and the late postprandial hypoglycemic attacks.^30^ There is a continuous on–off binding process in which the rate of association of antibodies with insulin is very fast and a higher association constant favors the bound fraction. The affinity of insulin for its tissue receptors may be a key determinant of the unbound fraction. In a study of the buffering effect of anti-insulin antibodies in a case of insulin autoimmune syndrome in which the antibody kinetics indicated that the antibody was monoclonal, in the absence of any overt monoclonal serum protein, the patient’s hypoglycemic episodes were so frequent and severe that treatment with high-dose glucocorticoids and subsequently plasmapheresis was required to try to decrease the antibody titer.^46^ Each approach lowered insulin antibody concentration, eliminated severe hypoglycemia, and improved insulin bioavailability and biodistribution, abolishing severe hypoglycemic episodes.

Any difference in the effects of a polyclonal versus a monoclonal anti-insulin antibody response in determining the duration of the insulin autoimmune syndrome has not been described. In Japan, where the disease has been studied systematically, the frequency of cases with a monoclonal anti-insulin autoantibody is very low (≤5% of cases). In persons of European descent, a monoclonal anti-insulin antibody response has been reported with a somewhat greater frequency, but the total number of cases of this rare syndrome in European or North American countries has been about one-tenth that of East Asian cases. Hence, studies of differential effects of antibody type in patients of European descent have not been reported.

The monoclonal anti-insulin response in persons who develop insulin autoimmune syndrome has not been linked with monoclonal gammopathy. There are no systematic data to suggest that cases of the former may evolve into that of clinical monoclonal gammopathy, and, in some cases, explicit evidence of normal immunoglobulin levels have been reported. The relationship between patients with the insulin autoimmune syndrome and a presumptive monoclonal antibody to insulin and those with monoclonal gammopathy and an immunoglobulin that has an affinity for insulin, if any, is not known to us. This statement excludes the one case reported in 2012 in which circumstantial evidence suggested that an insulin autoimmune syndrome evolved into myeloma with an anti-insulin monoclonal immunoglobulin (Table 1).^10^ Presumably, in the great majority of cases, the clone of B lymphocytes resulting in the insulin autoimmune syndrome mediated by a monoclonal anti-insulin immunoglobulin is neither of sufficient size to engender a discernible effect on the serum immunoglobulin levels nor is it subject to clonal evolution, as is the clone underlying essential monoclonal gammopathy.^10^

## Abbreviations

HLA: human leukocyte antigens
Ig: immunoglobulin
*K**_a_*: affinity constant.
